# 3-Substituted Prolines: From Synthesis to Structural Applications, from Peptides to Foldamers †

**DOI:** 10.3390/molecules18022307

**Published:** 2013-02-19

**Authors:** Céline Mothes, Cécile Caumes, Alexandre Guez, Héloïse Boullet, Thomas Gendrineau, Sylvain Darses, Nicolas Delsuc, Roba Moumné, Benoit Oswald, Olivier Lequin, Philippe Karoyan

**Affiliations:** 1 Laboratoire des BioMolécules, Université Pierre et Marie Curie-Sorbonne Universités, UMR 7203 and FR 2769, Paris, 75005, France; 2 CNRS, UMR 7203, Paris, 75005, France; 3 Département de Chimie, École Normale Supérieure, Paris, 75005, France; 4 Laboratoire Charles Friedel, Ecole Normale Supérieure de Chimie de Paris, UMR 7223, 11 rue Pierre et Marie Curie, Paris, 75005, France; 5 CNRS, UMR 7223, Paris, 75005, France; 6 Genzyme Pharmaceuticals, Eichenweg 1, CH-4410 Liestal, Switzerland

**Keywords:** substituted proline, peptide, peptidomimetics, β-turn, PPII helix

## Abstract

Among the twenty natural proteinogenic amino acids, proline is unique as its secondary amine forms a tertiary amide when incorporated into biopolymers, thus preventing hydrogen bond formation. Despite the lack of hydrogen bonds and thanks to conformational restriction of flexibility linked to the pyrrolidine ring, proline is able to stabilize peptide secondary structures such as β-turns or polyproline helices. These unique conformational properties have aroused a great interest in the development of proline analogues. Among them, proline chimeras are tools combining the proline restriction of flexibility together with the information brought by natural amino acids side chains. This review will focus on the chemical syntheses of 3-substituted proline chimeras of potential use for peptide syntheses and as potential use as tools for SAR studies of biologically active peptides and the development of secondary structure mimetics. Their influence on peptide structure will be briefly described.

## 1. Introduction

**Figure 1:**
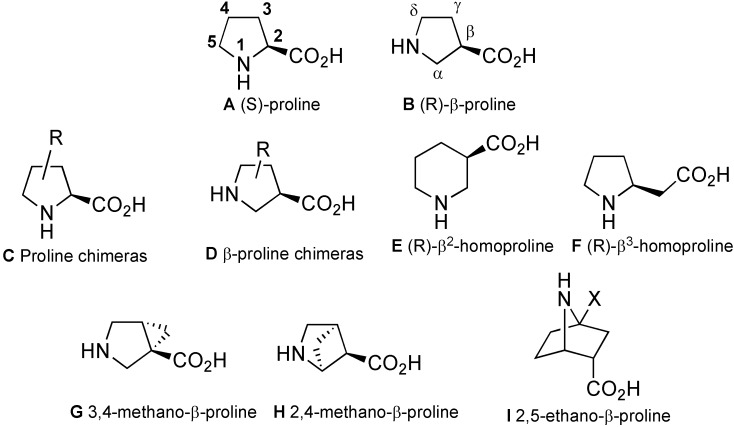
Some proline analogues.

Among the twenty natural amino acids, proline is unique as its secondary amine forms a tertiary amide when incorporated into biopolymers, thus preventing hydrogen bond formation. Regarding the conformational space around proline residues, if the pyrrolidine ring restricts the flexibility of Φ and ψ peptide backbone dihedral angles, at the same time the formed tertiary amide bond is more susceptible to *cis*/*trans* isomerism extending the accessible conformational space around the ω dihedral angle [1] Despite the lack of hydrogen bond donor capability, proline is able to stabilize peptide secondary structures such as turns [2] or helices (PPI and PPII helices) [3,4]. These unique conformational properties of proline have aroused a great interest leading to the development of many analogues (Figure 1) useful for peptide syntheses, SAR studies, design of bioactive peptides or secondary structures mimics [5,6,7,8,9,10,11,12].

**Figure 2:**
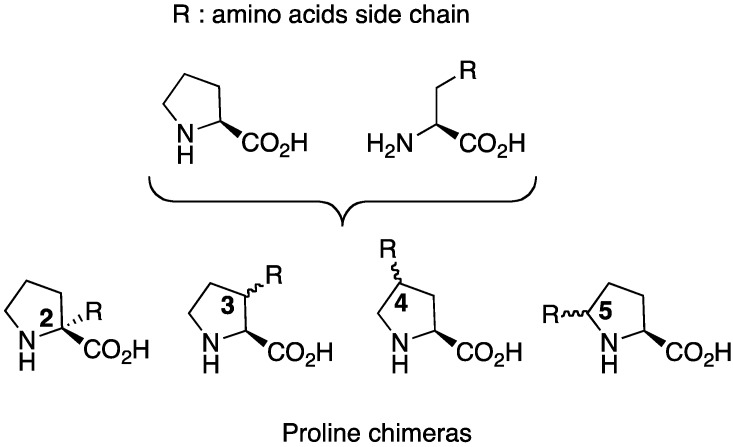
Four types of proline chimeras.

Among these analogues, proline chimeras **C** [5] are tools combining proline restriction of backbone flexibility together with the information brought by natural amino acids side chains. Four types of proline chimeras can be considered depending on the position of the side chain on the pyrrolidine ring (Figure 2). These cyclic amino acids restrain, like proline, the Φ-value of the peptide backbone around –65° while the insertion of side chains on the pyrrolidine ring concomitant with the creation of a new chiral centre might yield information on: (i) its conformation, (ii) the importance of the information it carries, (iii) the *cis*/*trans* isomerism of peptide amide bond. Among these chimeras, 3-substituted prolines have received careful attention regarding syntheses, structural features and biological applications, the subjects of this review.

## 2. Syntheses

The synthesis of mono-3-substituted prolines has been approached through several pathways that can be merged into three major routes involving direct functionalization of proline derivatives, inter- or intramolecular cyclization reactions (via C–C or C–N bond formation, using anionic, cationic or radicalar processes). Some of these approaches will be described here.

### 2.1. Syntheses Starting from Proline or Proline Derivatives

**Scheme 1:**
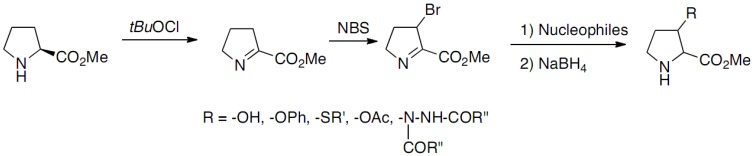
Nucleophilic substitution (NS) of bromine intermediate.

**Scheme 2:**
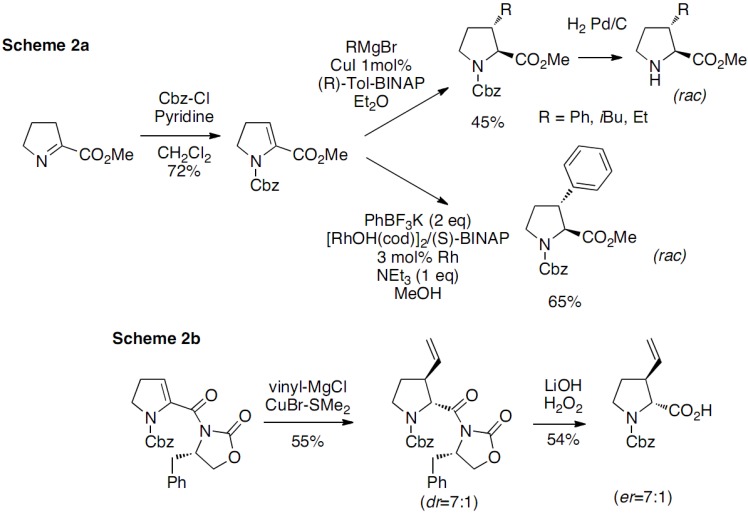
*Trans*-3-substituted prolines through 1,4-addition of organometallic reagents to 2,3-didehydroprolinate.

The synthesis of 3-substituted prolines through nucleophilic substitution (NS) of bromine intermediate (Scheme 1) was reported by Häusler and Schmidt in 1979 [13,14]:

The imine intermediate was obtained by *N*-chlorination of proline methyl ester with *tert*-butyl hypochlorite followed by dehydrochlorination, the oxidation leading to the loss of the chirality. The bromination of the imine intermediate was realized with *N*-bromosuccinimide and substitutions were performed with a few nucleophiles. The *substituted prolines were obtained as mixtures of cis/trans racemates. This approach has been extended in 2009 by Mothes*, who developed an original strategy based on the 1,4-addition of organometallic reagents to 2,3-didehydroprolinate (Scheme 2a) [15]:

The Michael acceptor was prepared from L-methylprolinate following Haüsler’s method [13]. Treatment of the iminoester with benzyl chloroformate in the presence of pyridine gave the desired *N*-protected 2,3-didehydroprolinate derivatives. 

Michael addition reactions were conducted first with Grignard reagents using 1 mol% of CuI and 1,5 mol% of the chiral ligand (*R*)-Tol-BINAP leading to the desired 1,4 adduct with excellent diastereoselectivity (dr > 99/1), but with no enantioselectivity. The stereochemical outcome of the addition was determined by the analysis of ROE correlations and vicinal coupling constants of the deprotected substituted proline, together with molecular mechanics calculations of *cis*- and *trans*-3-phenylproline isomers confirming the exclusively *trans* stereochemistry of the Michael adduct, obtained as a racemate. The rhodium-catalyzed 1,4-additions of phenyltrifluoroborate were also investigated in the presence of different Rh(I) catalysts and solvents. The reaction required the use of [RhOH(cod)]_2_ at high reaction temperatures to ensure the consumption of the starting material and affording 65% yield when conducted in MeOH. Again, attempts to develop a catalytic enantioselective reaction failed when a complex of [RhOH(cod)]_2_ associated with (*S*)-BINAP was used. The configuration of the Rh-catalyzed addition products was established by analogy with those from Cu-mediated addition.

A similar approach, but with improved yields regarding both the synthesis of the starting material and the 1,4 Michael addition on the enone, has been recently reported by Huy and co-workers [16]. Moreover, Huy succeeded elegantly in the synthesis of non racemic compounds (Scheme 2b) by introducing Evans chiral auxiliary on the dehydroproline derivative. However, the diastereoselective approach has only been described with vinyl Grignard reagent and it extension to other Grignard reagents was not demonstrated.

**Scheme 3:**
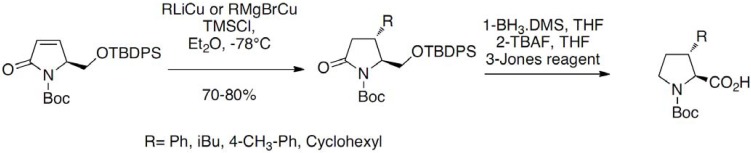
Addition of organocuprates on an enone derivative.

**Scheme 4:**
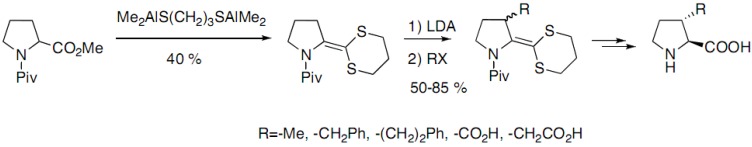
Regioselective alkylation of the allylic anion of the ketene *S,S*-acetal.

More recently, the Michael addition of organocuprates on an enone derivative has been successfully used by Maillard *et al.* for the stereoselective synthesis of 3-alkyl-substituted prolines (Scheme 3) [17]:

The starting enone was prepared in six steps from glutamic acid. The addition was performed in presence of TMSCl leading to the expected compounds in good yields (70%–80%) in all cases. Reduction of the pyrrolidine amide and TBAF deprotection of the silylated alcohol followed by Jones oxidation gave the desired derivatives as enantiomerically pure compounds. 3-Substituted prolines have been prepared as racemates by regioselective alkylation of the allylic anion of the ketene *S,S*-acetal derived from proline as reported by Moss and co-workers (Scheme 4) [18]:

*trans*-Derivatives were obtained as major product after regeneration of the carboxylic acid function. A possible interest in this approach compared to the previous ones is the introduction of functionalized side chains instead of simple alkyl chains.

**Scheme 5:**
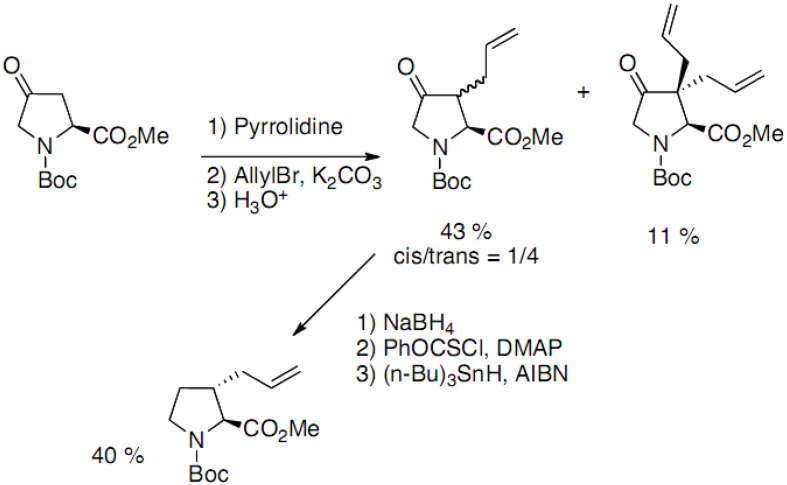
Regioselective alkylation of 4-oxoproline derivatives.

4-Oxoproline has been used by several groups as starting material to access 3-substituted prolines. In the method developed by Holladay, the regioselective C-3 alkylation is performed on an enamine [19]. A separable mixture of diastereoisomers is obtained, along with some dialkylation product (Scheme 5):

The introduction of the 9-phenylfluoren-9-yl instead of the Boc group allowed the direct regioselective alkylation of the ketone derivative as reported by Sharma [20].

**Scheme 6:**
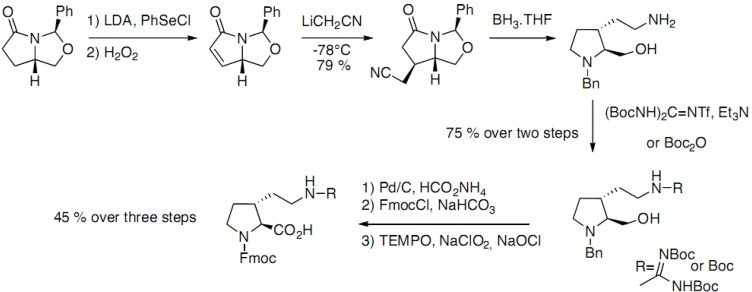
Diastereoselective addition of LiCH_2_CN on an α,β-unsaturated lactam.

Mamai *et al.* have reported the synthesis of optically pure *trans*-3-substituted prolines [21]. The strategy is based on the diastereoselective conjugate addition of LiCH_2_CN on an α,β-unsaturated lactam, obtained from (*S*)-pyroglutamic acid (Scheme 6).

The *trans*-substituted pyrrolidine was obtained after reduction of the lactam giving access to both amino acids suitably protected for peptide synthesis.

**Scheme 7:**
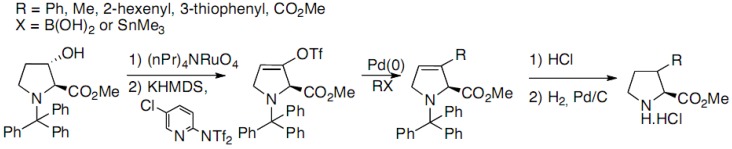
Palladium coupling on an enol triflate derived from 3-hydroxyproline.

**Scheme 8:**
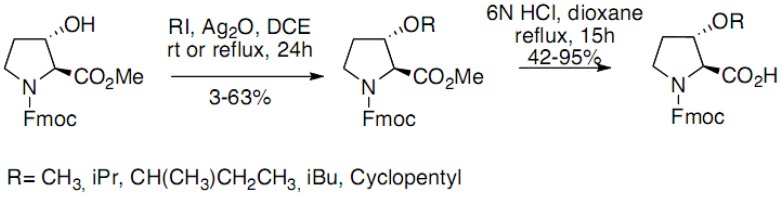
Alkylation of the alcohol function of 3-Hydroxyproline derivative.

3-Hydroxyproline has also used as a starting material for the synthesis of 3-substituted proline derivatives by some authors. Kamenecka has, for example, reported the palladium coupling on an enol triflate derived from 3-hydroxyproline (Scheme 7) [22]:

The enantioselective synthesis of 3-substituted prolines was achieved starting from 3-hydroxy-(*S*)-2-proline. A variety of groups were introduced at C3 position using palladium-mediated couplings with the corresponding enol triflate derived from *N*-trityl-3-oxo-(*S*)-2-proline methyl ester. Cleavage of the trityl residue and hydrogenation provided final products with good to modest diastereoselectivity (*cis-trans* mixtures). 3-Hydroxyproline has also been functionalized through simple alkylation of the alcohol function by alkyliodides by Maillard *et al.* (Scheme 8) [17]:

The Ag_2_O mediated alkylation led to proline derivatives with low to good yields (3%–63%). The acid mediated hydrolysis of the esters afforded, with moderate to good yields, derivatives suitable for peptide syntheses.

### 2.2. Syntheses by Intramolecular Cyclization Processes

#### 2.2.1. C–C Bond Formation through Anionic Processes

**Scheme 9:**
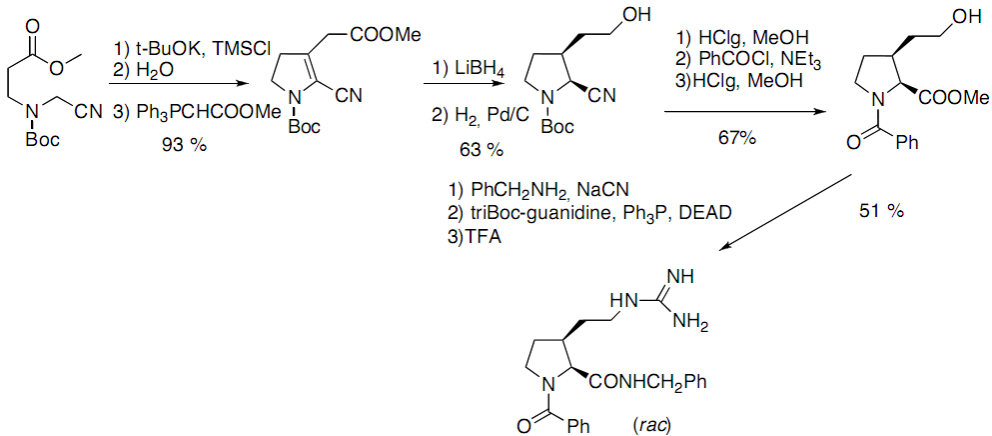
Intramolecular cyclization of a linear nitrile derivative.

Pellegrini and co-workers have reported a racemic synthesis of *cis-* and *trans-*3*-*substituted prolines functionalized by a guanidinoethyl group (Scheme 9) [23]. Addition of the carbanion on the ester, trapping of the enol by TMSCl followed by hydrolysis and Wittig reaction yielded the Boc-protected 3-pyrrolidinone:

The *cis* derivative is then obtained after catalytic hydrogenation of the double bond. The thermodynamically more stable *trans* derivative can be obtained by epimerisation of the α-centre, after hydrolysis of the nitrile. 

**Scheme 10:**
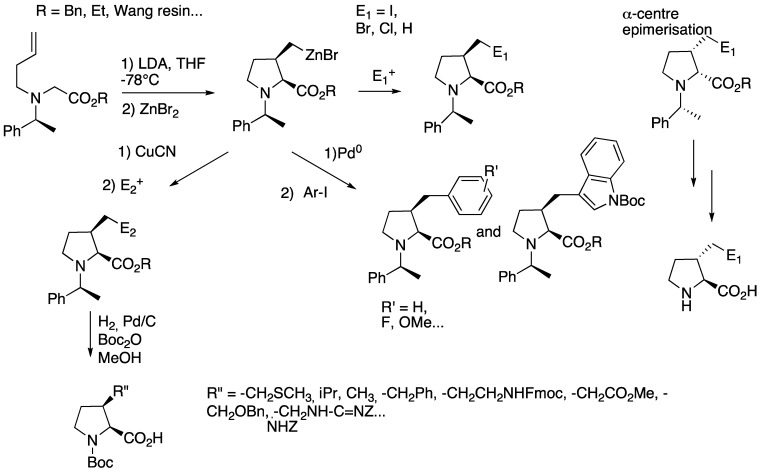
Amino-Zin-Ene-Enolate cyclization.

In 1997, Karoyan [24] and Lorthiois [25] reported the amino-zinc-ene-enolate cyclization as a powerful approach for the synthesis of substituted prolines. This reaction was applied to the synthesis of 3-substituted prolines bearing all types of natural amino acids side chains (Scheme 10) [26,27,28]. Starting from the *N*-homoallyl-α-amino benzylester [29], the intramolecular carbometallation yielded the cyclic organozinc reagent with a *cis*-stereochemistry. The reaction is highly stereospecific and stereoselective, the absolute configuration depending on the chiral auxiliary (*i.e.*, (*S)*- or (*R)*-α-methylbenzylamine). *trans*-Isomers were obtained by epimerization of the α-centre, the condition of epimerization depending on the side chain type.

The cyclic organozinc reagent was then reacted with electrophiles (NIS, NBS, I_2_, H_2_O) or transmetallated into palladium- or copper-zinc species to introduce variable functional groups. Noticeably, analogues of tryptophan were prepared on large scale through this strategy.

#### 2.2.2. C–C Bond Formation through Cationic Process

**Scheme 11:**
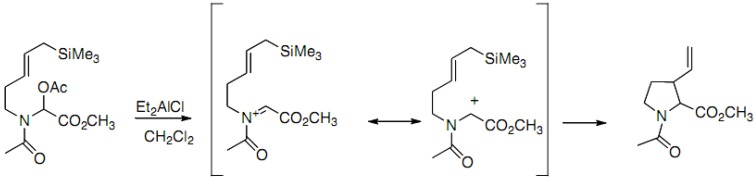
Intramolecular cyclization of *N*-acyliminium cation.

In 1987, the synthesis of racemic proline derivatives by intramolecular cyclization of propargyl- or allylsilane on *N*-acyliminium cation was reported by Mooiver (Scheme 11) [30]:

The 2-aza-Cope rearrangement usually observed in this approach is avoided thanks to the allylsilane double bond activation.

#### 2.2.3. C–C Bond Formation through Radicalar Process

**Scheme 12:**
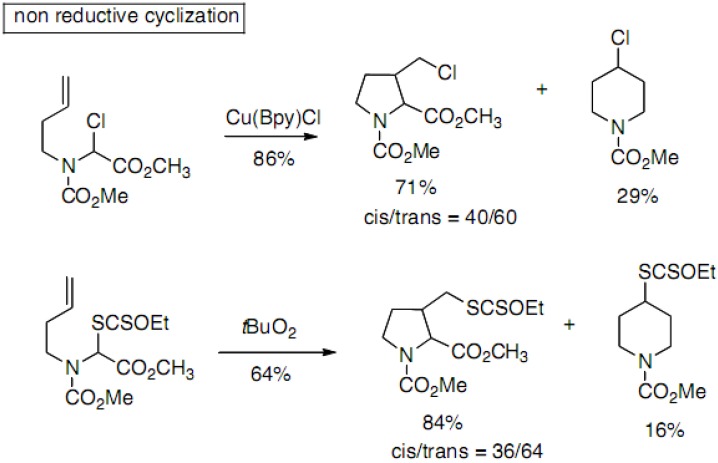
Cyclization through radical processes.

Hiemstra and co-workers have reported the synthesis of 3-substituted prolines with reductive and non-reductive radical cyclization processes [31,32,33], the former one allowing further functionalization. Compounds are obtained as mixtures of five- and six-membered rings (Scheme 12).

#### 2.2.4. C–N Bond Formation

##### 2.2.4.1. From Aspartic or Glutamic Acid Derivatives

**Scheme 13:**
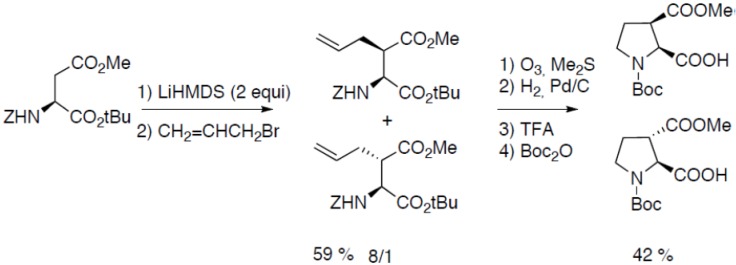
Aspartic acid as starting material.

Starting from orthogonally protected aspartic acid, North and co-workers have reported the synthesis of *cis-* and *trans-*3-carboxyproline derivatives (Scheme 13) [34]:

The α-proton abstraction was avoided by using the hindered base LiHMDS. The regioselective alkylation of the β position was performed on the dianion [35]. Oxydation of the double bond in reductive conditions followed by hydrogenation of the cyclic imine intermediate yielded the orthogonally protected amino acids.

##### 2.2.4.2. From Garner’s Aldehyde

**Scheme 14:**
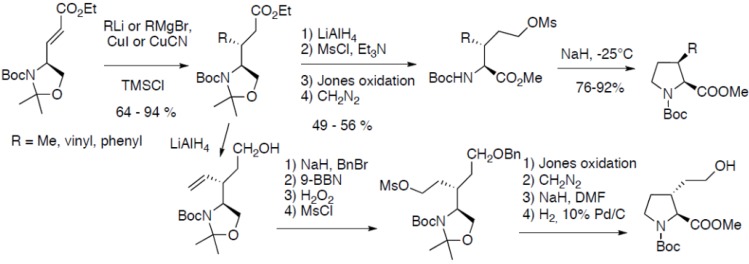
Diastereoselective 1,4-addition on a chiral oxazolidine α,β-unsaturated ester.

There are several contributions from Sasaki and co-workers reporting the syntheses of substituted prolines. Thus, the diastereoselective synthesis of *cis*-3-methyl-, vinyl- and phenylprolines have been reported (Scheme 14) [36]: 

The key step is the highly diastereoselective 1,4-addition of dialkylcuprates on a chiral oxazolidine α,β-unsaturated ester easily available from Garner’s aldehyde. The resulting linear precursor leads to the corresponding *cis*-3-substituted prolines after cyclization of the intermediate mesylate. This approach has been more recently extended to the synthesis of *trans*-derivatives after reduction of the ester function, followed by benzylation of the resulting alcohol, subsequent oxidative hydroboration of the alkene, followed by mesylation of the resulting alcohol and cyclization. The cyclic proline derivative was used as starting material to prepare arginine derivatives in 12 steps starting from Garner’s aldehyde [37].

**Scheme 15:**
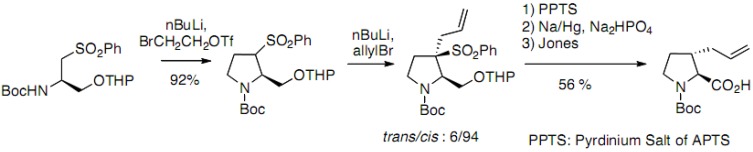
α-alkylation of a sulfone derived from serine.

An alternative approach has been reported for the synthesis of *trans*-derivatives based on the α-alkylation of a sulfone derived from serine (Scheme 15) [38]. The dianion generated with BuLi reacts with 2-bromoethyltriflate to allow the diastereoselective formation of the pyrrolidine cycle, which was subsequently alkylated by allylbromide. The thermodynamically more stable *trans* derivative was obtained with good optical purity after desulfonylation [38,39,40]. 

##### 2.2.4.3. Through Reductive Amination

**Scheme 16:**
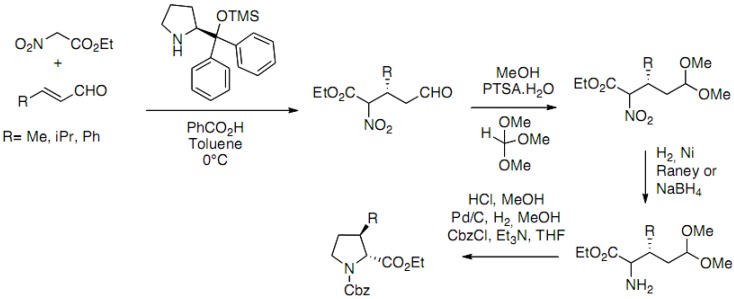
Asymmetric organocatalytic Michael addition of nitro esters.

An elegant enantioselective organocatalytic approach has been recently reported by Han *et al.* (Scheme 16) [41]:

The key step is an asymmetric organocatalytic Michael addition of nitro esters to α,β-unsaturated aldehydes. Chiral adducts are obtained with excellent yields and enantioselectivities. The diarylprolinol silyl ether is used as the organocatalyst. The optimal conditions reported are 10 mol% of the catalyst with 10 mol% of benzoic acid in toluene at 0 °C. After protection of the aldehyde function, reduction of the nitro group, the authors succeeded in the synthesis of the protected proline derivatives in a one pot procedure including acetal deprotection, imine formation followed by catalytic hydrogenation and Cbz protection of the amine.

### 2.3. Synthesis of 3-Substituted Prolines by Intermolecular Cyclization

#### 2.3.1. Through Michael-Addition/Alkylation Sequences

**Scheme 17:**
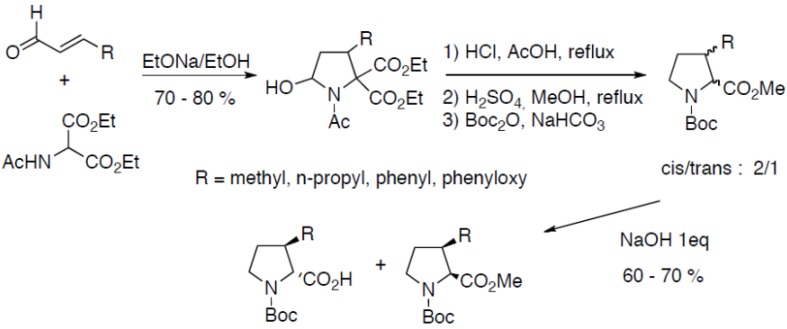
Condensation of diethyl *N*-acetylaminomalonate on α,β-unsaturated aldehydes.

3-Substituted prolines can also be prepared through the condensation of diethyl *N*-acetylaminomalonate on α,β-unsaturated aldehydes (Scheme 17) [42,43,44,45]:

Compounds are obtained as a mixture of *N*-Boc-proline diastereoisomers after three steps. The *cis-* and *trans-* derivatives were separated thanks to the selective saponification of the less hindered *trans-* derivative. Coupling the racemate to α-methylbenzylamine has also been described to obtain optically pure compounds [44]. The organocatalytic asymmetric version of this reaction has been reported by Rios *et al.* using chiral pyrrolidine derivatives as catalysts [46]. The organocatalytic enantioselective tandem reactions proved to be highly enantioselective and occured with good yields (67%–77%) and excellent *ee* (90%–99%).

## 3. Conformational Effects and Structural Applications

### 3.1. Conformational Effects of 3-Substituted Prolines

**Figure 3:**
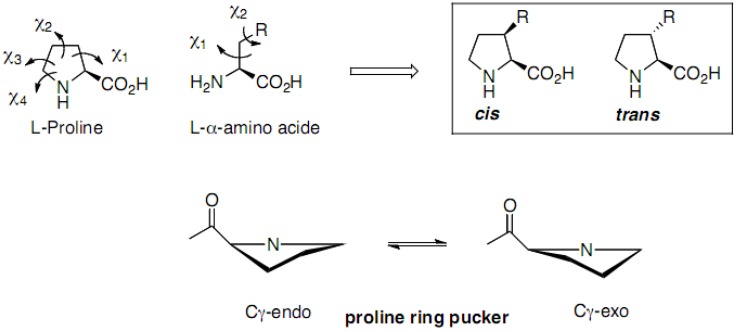
Schematic representation of *trans* and *cis*-3-prolinoamino acids.

The conformational effects of these proline chimeras strongly depend on different parameters from the nature and the bulkiness of the side chain to the configuration of the C3 carbon (Figure 3). The introduction of a methyl group with a *trans* relationship with the carboxyl function has only minor effects, whereas a *cis*-3-methyl susbstituent stabilizes the C^γ^-endo puckering and strongly restricts the conformational space around the ψ angle, through steric interactions with the carboxamide group, strongly destabilizing the γ-turn conformation for example [47]. 

Increasing the bulkiness of the *trans-*3-substituent (from methyl to isopropyl) gradually shifts the puckering equilibrium toward the Cγ-exo form (from 50% for *trans-*3-methylethiomethylproline to 70% for *trans-*3-isopropylproline) [48]. This Cγ-exo puckering of *trans-*3-isopropylproline corresponds to less negative values of Φ and smaller values of ψ as compared to proline.

Regarding the effects of a 3-substituent on *cis*-*trans* isomerism of the preceding peptide bond, 3-methyl substituents marginally affect the proportion of peptide bond *cis* and *trans* isomers (about 25%–30% of *cis* isomers in water, as observed for other peptides) [47,49].

The pyrrolidine cycle restricts the side chain conformational space since only two of the three possible χ1 canonical rotamers of unconstrained amino acids are accessible to prolinoamino acids, the *trans* and a single *gauche* rotamer (g^−^ or g^+^ for *trans* or *cis*-prolinoamino acids, respectively). Although the geometrical constraint due to the cyclization induces a 30° deviation of χ1 from ideal staggered values, the conformers of prolinoamino acids fit well with the corresponding structures of unconstrained amino acids [48,50].

### 3.2. Structural Applications

Despite the lack of hydrogen-bond donor ability when introduced into peptide sequences, the conformational restriction endows proline with a high propensity for secondary structures such as extended helices [4] or β-turns [2].

#### 3.2.1. Polyproline Helical Conformations

Proline-rich peptide sequences can fold into helical conformations: the polyproline II (PPII) helix with *trans* amide bonds in aqueous solvents or the polyproline I (PPI) helix with *cis* amide bonds in polar organic solvents. The PPII helix is often encountered in proteins such as collagen triple helix where the three strands are folded into PPII conformation [51,52]. In addition, such PPII motifs play important roles in protein-ligand or protein-protein recognition [4,53,54,55]. On the contrary, the PPI helix is not encountered in a biological context.

**Scheme 18:**
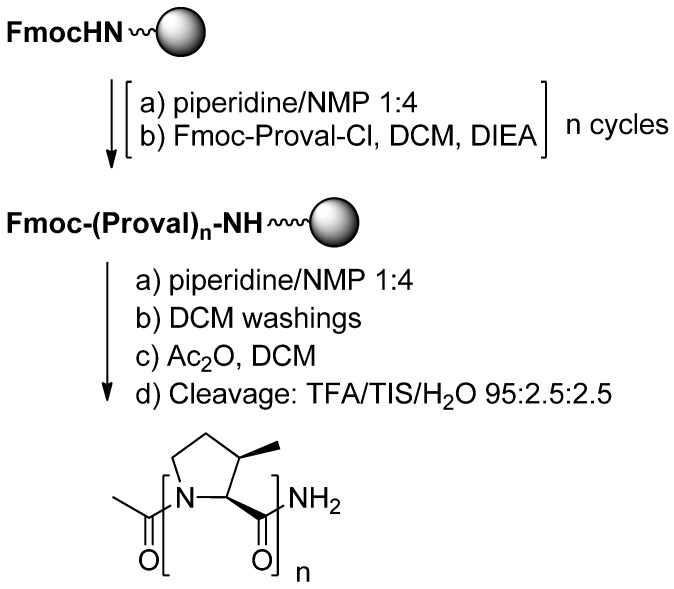
Solid phase synthesis of oligomers.

Functionalized PPII helices have been designed by using proline chimeras, such as 4-substituted prolines [3,56] and 3-substituted prolines [4]. In the latter case, a major difficulty is encountered during peptide synthesis. Indeed, the incorporation of *cis*- and *trans*-3-substituted prolines can be realized by using a panel of standard coupling reagents such as HBTU, HATU, DCC/DMAP, PyBrOP, DIC/HOBt with or without microwave activation but with only moderate yields in the case of *cis* derivatives. Because of steric hindrance of *cis*-3-substituted derivatives, the solid phase synthesis of oligomers requires an optimization of conditions of the coupling step, which can be driven to completion after activation through the corresponding acyl chlorides. This was demonstrated by the synthesis of prolinovaline oligomers (Scheme 18):

Initially, the required acyl chloride of prolinovaline monomer was prepared using SOCl_2_ into refluxing dichloromethane. The product was found to be stable when stored into desiccators after evaporation of the solvent, but reproducibility problems were encountered by using this method. Use of the Ghosez reagent solved this problem [57,58]. In this case, the reaction mixture was found to be directly utilizable for coupling onto the resin without removal of excess reagent and side product. Homooligomers of prolinovaline from two to nine residues were synthesized with Ghosez reagent with yields ranging from 11% to 59%. By contrast, during the solid phase synthesis of a peptide incorporating one or more prolinovaline residues, the amine group of the prolinovaline monomer reacts easily with natural α-amino acid using the standard HBTU coupling reagent.

**Figure 4:**
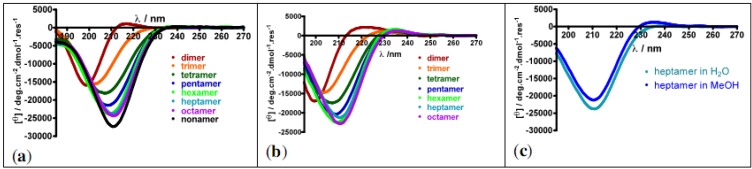
CD spectra of *cis*-prolinovaline oligomers (from dimer to octa- or nonamer) (**a**) in H_2_O; (**b**) in MeOH; (**c**) Superimposition of the heptamer CD spectra recorded in water and MeOH.

The structures of the prolinovaline oligomers were assessed using Circular Dichroism (CD, Figure 4). CD is a commonly used technique for rapid identification of secondary structures in proteins. It also allows getting insights in the folding propensity of non-natural oligomers or polyproline oligomers when other techniques do not. For example, PPII conformations are difficult to establish by NOE's NMR experiments since the extended conformation prevents the existence of spatial correlation (NOE) between non-adjacent residues. The existence of *cis*/*trans* interconversion equilibria also often hampers the interpretation of NMR spectra. 

Oligomers built from prolines adopt extended conformations in solution, *i.e.* PPII conformation in water and PPI conformation in aliphatic alcohols such as MeOH. In the case of polyproline conformations, characteristic CD signatures are observed: a weak maximum at 226 nm and a strong minimum at 206 nm in water for PPII conformation, and weak minima at 200 and 232 nm, together with a strong maximum at 215 nm in aliphatic alcohols for PPI conformation [59]. β-Structure/PPII/PPI helix interconversions have been studied using non-natural proline surrogates substituted in various positions [60,61,62,63,64]. 

From the prolinovaline hexamer, the CD spectra do not depict to the sum of the chiral contribution of each monomer but indicate a defined chiral secondary structure in water. Moreover, upon increasing the temperature from 20 to 90 °C, the shape of the CD signal remains unchanged, indicating that the secondary structure adopted by the prolinovaline oligomers is stable in this temperature range. Similar CD spectra were recorded in MeOH, and the CD spectra of the heptamer in water and MeOH (Figure 4c) are almost superimposable, suggesting that the PPII conformation is locked in both solvents. This indicates that the substitution in position 3 of the pyrrolidine ring can be accommodated in PPII secondary structures in water and even in aliphatic alcohol. Prolinoamino acids appear to be valuable tools to build functionalized foldamers mimetics of PPII helices.

#### 3.2.2. β-Turns

**Figure 5:**
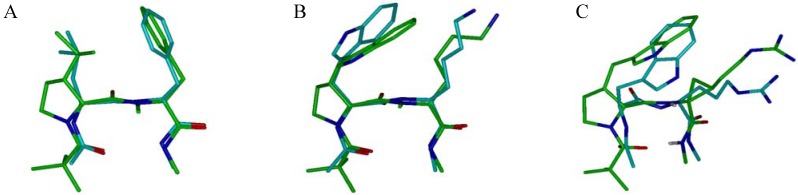
Comparison of turn mimetics NMR structures with selected β-turns from PDB structures. The turn mimetics are pseudotetrapeptides of sequence Piv-D-Xaa-L-Yaa-NHMe with Xaa = 3-*cis*-prolinoleucine (**A**) or 3-*cis*-prolinohomotryptophane (**B**, **C**) and Yaa = NMePhe (**A**), NMeLys (**B**) or cyclopropylarginine (**C**). The peptidomimetics structures are superimposed to the following structures: **A**, Leu-Phe β-turn of glycogen phosphorylase (PDB entry 3GPB); **B**, Trp-Lys β-turn of a somatostatin analog (PDB 1SOC); **C**, Trp-Arg β-turn of tendamistat (PDB 1BVN).

The β-turn motif, a recognition element often involved in receptor-ligand interactions [65], is a major subject of investigation in the development of synthetic mimics of peptide secondary structure [66,67,68,69,70,71,72,73,74,75,76]. The use of *cis*-3-substituted prolinoamino acids in combination with *N*-methyl- or cyclopropyl amino acids has been reported to stabilize type II’ β-turns in water that retain the side-chain functionalities in both i+1 and i+2 positions of the turn [50,68]. These short peptides incorporate three motifs, a heterochiral sequence, a proline scaffold and a *N*-methyl group or a cyclopropylamino acid, that are known to exhibit strong β-turn propensity (Figure 5) [1,73,74,75]. The prolinoamino acid allows to mimic the canonical staggered rotamers of the side chains in i+1 position with minimal deviation and its combination with *N*-methyl- or cyclopropylamino acids allows one to mimic the three canonical rotamers of the χ1 angle in position i+2 of the turn. Noticeably, the *N*-methylation restricts the conformational space of the side chain in i+2 position to *gauche*– (χ1 ~ –60°) and *trans* (χ1 ~ 180°) orientations around the χ^1^ angle, the *gauche*+ rotamer (χ1 ~ +60°) being destabilized by unfavorable interaction with the *N*-methyl group. Heterochiral sequences incorporating the side chains of aromatic hTrp and cationic Lys or Arg amino acids were prepared as secondary structure mimics of the turn sequences found in somatostatin [77] and tendamistat respectively (Figure 5) [78].

## 4. Conclusions

In conclusion, 3-substituted prolines have been reported as useful tools for SAR studies. They also represent powerful tools to build stable functionalized secondary structure mimetics, such as turns or PPII helices, a primary step towards the design of more sophisticated foldamers. However, reaching such goals requires the development of synthetic methodologies allowing the preparation of these susbtituted prolines with high functional diversity. Several strategies have been reported, some of them allowing the multi-gram scale synthesis of these chimeras.

## References

[1] Wawra S., Fischer G., Dugave E.C. (2006). Amide *cis-trans* Isomerisation in Peptides and Proteins. Cis-Trans Isomerization in Biochemistry.

[2] Aubry A., Vitoux B., Marraud M. (1985). Conformational properties of Pro–Pro sequences. I. Crystal structures of two dipeptides with L-Pro-L-Pro and L-Pro-D-Pro sequences. Biopolymers.

[3] Fillon Y.A., Anderson J.P., Chmielewski J. (2005). Cell Penetrating agents based on a polyproline helix scaffold. J. Am. Chem. Soc..

[4] Jacquot Y., Broutin I., Miclet E., Nicaise M., Lequin O., Goasdoué N., Joss C., Karoyan P., Desmadril M., Ducruix A., Lavielle S. (2007). High affinity Grb2-SH3 domain ligand incorporating C^β^-substituted prolines in a Sos-derived decapeptide. Bioorg. Med. Chem..

[5] Karoyan P., Sagan S., Lequin O., Quancard J., Lavielle S., Chassaing G., Attanasi O.A., Spinelli D. (2004). Susbtituted Prolines: Syntheses and Applications in Structure-Activity Relationship Studies of Biologically Active Peptides. Targets in Heterocyclic Systems-Chemistry and Properties.

[6] Huck B.R., Fisk J.D., Guzei I.A., Carlson H.A., Gellman S.H. (2003). Secondary structural preferences of 2,2-disubstituted pyrrolidine-4-carboxylic acid oligomers: β-Peptide foldamers that cannot form internal hydrogen bonds. J. Am Chem. Soc..

[7] Huck B.R., Gellman S.H. (2005). Synthesis of 2,2-disubstituted pyrrolidine-4-carboxylic acid derivatives and their incorporation into β-peptide oligomers. J. Org. Chem..

[8] Medda A.K., Lee H.-S. (2009). 3,4-Methano-β-proline: A conformationally constrained β-amino acid. Synlett.

[9] Krow G.R., Liu N., Sender M., Lin G., Centafont R., Sonnet P.E., DeBrosse C., Ross C.W., Carroll P.J., Shoulders M.D., Raines R.T. (2010). Oligomers of a 5-carboxy-methanopyrrolidine-amino acid. A search for order. Org. Lett..

[10] Otani Y., Futaki S., Kiwada T., Sugiura Y., Muranaka A., Kobayashi N., Uchiyama M., Yamaguchi K., Ohwada T. (2006). Oligomers of β-amino acid bearing non-planar amides form ordered structures. Tetrahedron.

[11] Hosoya M., Otani Y., Kawahata M., Yamaguchi K., Ohwada T. (2010). Water-stable helical structure of tertiary amides of bicyclic -amino acid bearing 7-azabicyclo[2.2.1]heptane. Full control of amide cis-trans equilibrium by bridgehead substitution. J. Am. Chem. Soc..

[12] Abele S., Vögtli K., Seebach D. (1999). Oligomers of β^2^- and of β^3^-Homoproline: What are the secondary structures of β-peptides lacking H-Bonds?. Helv. Chim. Acta.

[13] Häusler J., Schmidt U. (1979). Synthese von *cis*- und *trans*-3-Phenoxyprolin. Liebigs Ann. Chem..

[14] Häusler J. (1981). Darstellung von *cis*- und *trans*-C-3-substituierten Prolinverbindungen. Liebigs Ann. Chem..

[15] Mothes C. Prolino Amino Acides Substitués en Position 3. Synthèses, Applications Structurales et Pharmacologiques dans le Développement D'inhibiteurs D'interactions Peptide-Protéine et Protéine-Protéine. Ph.D. Thesis.

[16] Huy P., Neudörfl J.-M., Schmalz H.-G. (2011). A practical synthesis of trans-3-substituted proline derivatives through 1,4-addition. Org. Lett..

[17] Maillard M.C., Brookfield F.A., Courtney S.M., Eustache F.M., Gemkow M.J., Handel R.K., Johnson L.C., Johnson P.D., Kerry M.A., Krieger F. (2011). Exploiting differences in caspase-2 and -3 S2 subsites for selectivity: Structure-based design, solid-phase synthesis and *in vitro* activity of novel substrate-based caspase-2 inhibitors. Bioorg. Med. Chem..

[18] Moss W.O., Jones A.C., Wisedale R., Mahon M.F., Molloy K.C., Bradbury R.H., Hales N.J., Gallagher T. (1992). 2-Amino ketene S,S-acetals as a-amino acid homoenolate equivalents. Synthesis of 3-substituted prolines and molecular structure of 2-(*N*-pivaloylpyrrolidin-2-ylidene)-1.3-dithiane. J. Chem. Soc. Perkin Trans. 1.

[19] Holladay M.W., Lin C.W., May C.S., Garvey D.S., Witte D.G., Miller T.R., Wolfram C.A.W., Nadzan A.M. (1991). *trans*-3-*n*-Propyl-L-proline is a highly favorable, conformationally restricted replacement for methionine in the C-terminal tetrapeptide of cholecystokinin. Stereoselective synthesis of 3-allyl- and 3-n-propyl-l-proline derivatives from 4-hydroxy-l-proline. J. Med. Chem..

[20] Sharma R., Lubell W.D. (1996). Regioselective enolization and alkylation of 4-oxo-*N*-(9-phenylfluoren-9-yl)proline:  Synthesis of enantiopure proline−valine and hydroxyproline−valine chimeras. J. Org. Chem..

[21] Mamai A., Hughes N.E., Wurthmann A., Madalengoitia J.S. (2001). Synthesis of conformationally constrained arginine and ornithine analogues based on the 3-substituted pyrrolidine framework. J. Org. Chem..

[22] Kamenecka T.M., Park Y.-J., Lin L.S., Lanza T.J., Hagmann W.K. (2001). Enantioselective approach to 3-substituted prolines. Tetrahedron Lett..

[23] Pellegrini N., Schmitt M., Guery S., Bourgignon J.-J. (2002). New strategies towards proline derivatives as conformationally constrained arginine analogues. Tetrahedron Lett..

[24] Karoyan P., Chassaing G. (1997). New strategy for the synthesis of 3-substituted prolines. Tetrahedron Lett..

[25] Lorthiois E., Marek I., Normant J.-F. (1997). Zinca-ene-allene and zinc-enolate cyclization. Towards the synthesis of polysubstituted pyrrolidines. Tetrahedron Lett..

[26] Karoyan P., Quancard J., Vaissermann J., Chassaing G. (2003). Amino-Zinc-Enolate carbometalation reactions: Application to ring closure of terminally substituted olefin for the asymmetric synthesis of *cis*- and *trans*-3-prolinoleucine. J. Org. Chem..

[27] Quancard J., labonne A., Jacquot Y., Lavielle S., Chassaing G., Karoyan P. (2004). Asymmetric synthesis of 3-substituted proline chimeras bearing polar side chains of proteinogenic amino acids. J. Org. Chem..

[28] Mothes C., Lavielle S., Karoyan P. (2008). Amino-zinc-ene-enolate cyclization: A short access to *cis*-3-substituted prolino-homotryptophane derivatives.

[29] Amine commercially available from Genzyme, Eichenweg 1, CH-4410 Liestal Switzerland, Tel.: +41-(0)61-906-5959 Fax: +41-(0)61-906-5958.

[30] Mooiver H.H., Hiemstra H., Fortgens H.P., Speckamp W.N. (1987). Intramolecular reactions of acyclic n-acyliminium ions III silicon assisted cyclocondensation of glyoxylic esters to proline and pipecolic acid derivatives. Tetrahedron Lett..

[31] Esch P.M., Hiemstra H., Speckamp W.N. (1990). Synthesis of cyclic α-amino acids through ring closure of glycine derived free radicals. Tetrahedron Lett..

[32] Udding J.H., Giesselink J.P.M., Hiemstra H., Speckamp W.N. (1994). Xanthate transfer cyclisation of glycine radicals; synthesis of 5- and 6-membered ring nitrogen heterocycles. Bull. Soc. Chim. Belg..

[33] Udding J. H., Tuijp J.M., Hiemstra H., Speckamp W.N. (1994). Transition metal-catalyzed chlorine transfer cyclizations of carbon-centered glycine radicals; a novel synthesis route to cyclic α-amino acids. Tetrahedron.

[34] Cotton R., Johnstone A.N.C., North M. (1995). Asymmetric synthesis of 3-carboxyproline and derivatives suitable for peptide synthesis. Tetrahedron.

[35] Baldwin J.E., Moloney M.G., North M. (1989). Asymmetric amino acid synthesis: preparation of the β anion derived from aspartic acid. J. Chem. Soc. Perkin Trans. 1.

[36] Flamant-Robin C., Wang Q., Chiaroni A., Sasaki A. (2002). An efficient method for the stereoselective synthesis of cis-3-substituted prolines: Conformationally constrained α-amino acids. Tetrahedron.

[37] Kumar S., Wang Q., Sasaki A. (2007). Synthesis of conformationally constrained analogues of RGD tripeptide. Tetrahedron.

[38] Sasaki A., Hashimoto C., Chiaroni A., Riche C., Potier P. (1987). A novel approach to the synthesis of optically pure non protein α-amino acids in both L and D configurations from L-serine. Tetrahedron Lett..

[39] Sasaki A., Dockner M., Chiaroni A., Riche C., Potier P. (1997). A novel stereodivergent synthesis of optically pure cis- and trans-3-substituted proline derivatives. J. Org. Chem..

[40] Sasaki A., Pauly R., Fontaine C., Chiaroni A., Riche C., Potier P. (1994). Enantioselective synthesis of (2*S*,3*S*)- and (2*R*,3*R*)-pyrrolidine-2,3-dicarboxylic acids: Conformationally constrained (*S*)- and (*R*)-aspartic acid analogues. Tetrahedron Lett..

[41] Han M.-Y., Zhang Y., Wang H.-Z., An W.-K., Ma B.-C., Zhang Y., Wanga W. (2012). Organocatalytic michael addition of nitro esters to a,b-unsaturated aldehydes: Towards the enantioselective synthesis of *trans*-3-substituted proline derivatives. Adv. Synth. Catal..

[42] Cox D.A., Johnson A.W., Mauger A.B. (1964). A modified proline synthesis. J. Chem. Soc..

[43] Mosberg H.I., Omnaas J.R., Lomize A., Heyl D.L., Nordan I., Mousigian C., Davis P., Porreca F. (1994). Development of a model for the δ opioid receptor pharmacophore. 1. Conformationally restricted Tyr1 replacements in the cyclic δ receptor selective tetrapeptide Tyr-c[D-Cys-Phe-D-Pen]OH (JOM-13). J. Med. Chem..

[44] Chung J.Y.L., Wasicak J.T., Arnold W.A., May C.S., Nadzan A.M., Holladay M.W.  (1990). Conformationally constrained amino acids. Synthesis and optical resolution of 3-substituted proline derivatives. J. Org. Chem..

[45] Damour D., Doerflinger G., Pantel G., Labaudinière R., Leconte J.-P., Sablé S., Vuilhorgne M., Mignani S. (1999). A convenient synthetic route to macrocyclic *cis*-3-phenylproline derivatives as mimics of sandostatin®. Synlett.

[46] Rios R., Ibrahem I., Vesely J., Sundén H., Cordova A. (2007). Organocatalytic asymmetric 5-hydroxypyrrolidine synthesis: A highly enantioselective route to 3-substituted proline derivatives. Tetrahedron Lett..

[47] Delaney N.G., Madison V. (1982). Novel conformational distributions of methylproline peptides. J. Am. Chem. Soc..

[48] Quancard J., Karoyan P., Lequin O., Wenger E., Aubry A., Lavielle S., Chassaing G. (2004). Prolinoamino acids as a tool to stabilize β-turns with the side chain of natural amino acids. Tetrahedron Lett..

[49] Beausoleil E., Sharma R., Michnick S.W., Lubell W.D. (1998). Alkyl 3-position substituents retard the isomerization of prolyl and hydroxyprolyl amides in water. J. Org. Chem..

[50] Mothes C., Larregola M., Quancard J., Goasdoué N., Lavielle S., Chassaing G., Lequin O., Karoyan P. (2010). Prolinoamino acids as tools to build bifunctionalized, stable β-turns in water. ChemBioChem..

[51] Brodsky B., Thiagarajan G., Madhan B., Kar K. (2008). Triple-helical peptides: An approach to collagen conformation, stability, and self-association. Biopolymers.

[52] Shoulders M.D., Raines R.T. (2009). Collagen structure and stability. Annu. Rev. Biochem..

[53] Rabanal F., Ludevid M.D., Pons M., Giralt E. (1993). CD the of proline-rich polypeptides: Application to the study of repetitive domain of maize glutelin-2. Biopolymers.

[54] Kay B.K., Williamson M.P., Sudol M. (2000). The importance of being proline: the interaction of proline-rich motifs in signaling proteins with their cognate domains. FASEB J..

[55] Williamson M.P. (1994). The structure and function of proline-rich regions in proteins. Biochem. J..

[56] Kalafut D., Anderson T.N., Chmielewski J. (2012). Mitochondrial targeting of a cationic amphiphilic polyproline helix. Bioorg. Med. Chem. Lett..

[57] Caumes C., Delsuc N., Beni Azza R., Correia I., Chemla F., Ferreira F., Carlier L., Perez Luna A., Moumné R., Lequin O. (2013). Homooligomers of substituted proline and β-prolines: Synthesis and secondary structure investigation by CD experiments. New J. Chem..

[58] Devos A., Remion J., Frisque-Hesbain A.-M., Colens A., Ghosez L. (1979). Synthesis of acyl halides under very mild conditions. J. Chem. Soc. Chem. Commun..

[59] Kümin M., Sonntag L.-S., Wennemers H. (2007). Azidoproline containing helices: Stabilization of the polyproline II structure by a functionalizable group. J. Am. Chem. Soc..

[60] Sonar M.V., Ganesh K.N. (2010). Water-induced switching of β-structure to polyproline II conformation in the 4*S*-aminoproline polypeptide via H-bond rearrangement. Org. Lett..

[61] Zhang R., Brownewell F., Madalengoitia J.S. (1998). Pseudo-A(1,3) Strain as a key conformational control element in the design of poly-L-proline type II peptide mimics. J. Am. Chem. Soc..

[62] Kuemin M., Nagel Y.A., Schweizer S., Monnard F.W., Ochsenfeld C., Wennemers H. (2010). Tuning the *cis/trans* conformer ratio of Xaa–Pro amide bonds by intramolecular hydrogen bonds: The effect on PPII helix stability. Angew. Chem. Int. Ed..

[63] McCafferty D.G., Friesen D.A., Danielson E., Wall C.G., Saderholm M.J., Erickson B.W., Meyer T.J. (1996). Photochemical energy conversion in a helical oligoproline assembly. Proc. Natl. Acad. Sci. USA.

[64] Sagan S., Quancard J., Lequin O., Karoyan P., Chassaing G., Lavielle S. (2005). Conformational analysis of the C-Terminal Gly-Leu-Met-NH_2_ tripeptide of substance P bound to the NK-1 receptor. Chem. Biol..

[65] Tyndall J.D., Pfeiffer B., Abbenante G., Fairlie D.P. (2005). Over one hundred peptide-activated G protein-coupled receptors recognize ligands with turn structure. Chem. Rev..

[66] Seebach D., Abele S., Gademann K., Jaun B. (1999). Pleated sheets and turns of β-peptides with proteinogenic side chains. Angew. Chem. Int. Ed..

[67] Gademann K., Kimmerlin T., Hoyer D., Seebach D. (2001). Peptide folding induces high and selective affinity of a linear and small β-peptide to the human somatostatin receptor 4. J. Med. Chem..

[68] Guitot K., Larregola M., Pradhan T.K., Vasse J.-L., Lavielle S., Bertus P., Szymoniak J., Lequin O., Karoyan P. (2011). The combination of prolinoamino acids and cyclopropylamino acids leads to fully functionalized, stable β-turns in water. ChemBioChem.

[69] Ball J.B., Alewood P.F. (1990). Conformational constraints: Nonpeptide β-turn mimics. J. Mol. Recognit..

[70] Souers A.J., Ellman J.A. (2001). β-Turn mimetic library synthesis: Scaffolds and applications. Tetrahedron.

[71] Kee K.S., Jois S.D.S. (2003). Design of β-turn based therapeutic agents. Curr. Pharm. Des..

[72] Barbaras D., Gademann K. (2008). Stable β turns of tripeptides in water through cation–π interactions. ChemBioChem.

[73] Imperiali B., Moats R.A., Fisher S.L., Prins T.J. (1992). A conformational study of peptides with the general structure Ac-L-Xaa-Pro-D-Xaa-L-Xaa-NH_2_: Spectroscopic evidence for a peptide with significant β-turn character in water and in dimethyl sulfoxide. J. Am. Chem. Soc..

[74] Chalmers D.K., Marshall G.M. (1995). Pro-D-NMe-Amino Acid and D-Pro-NMe-Amino Acid: Simple, efficient reverse-turn constraints. J. Am. Chem. Soc..

[75] Takeuchi Y., Marshall G.M. (1998). Conformational analysis of reverse-turn constraints by *N*-methylation and *N*-hydroxylation of amide bonds in peptides and non-peptide mimetics. J. Am. Chem. Soc..

[76] Chatterjee B., Saha I., Raghothama S., Aravinda S., Rai R., Shamala N., Balaram P. (2008). Designed peptides with homochiral and heterochiral diproline templates as conformational constraints. Chem. Eur. J..

[77] Weckbecker G., Lewis I., Albert R., Schmid H.A., Hoyer D., Bruns C. (2003). Opportunities in somatostatin research: Biological, chemical and therapeutic aspects. Nat. Rev. Drug Discov..

[78] Wiegand G., Epp O., Huber R. (1995). The crystal structure of porcine pancreatic α-amylase in complex with the microbial inhibitor tendamistat. J. Mol. Biol..

